# Safety and performance of Hydros and Hydros-TA for knee osteoarthritis: a prospective, multicenter, randomized, double-blind feasibility trial

**DOI:** 10.1186/s12891-015-0513-6

**Published:** 2015-03-18

**Authors:** Robert J Petrella, Pieter J Emans, Julia Alleyne, Frank Dellaert, Dawn P Gill, Marcee Maroney

**Affiliations:** Aging, Rehabilitation & Geriatric Care Research Centre, Lawson Health Research Institute & Departments of Family Medicine, Kinesiology and Cardiology, Western University, London, Canada; Department of Orthopaedic Surgery, Maastricht University Medical Center, Maastricht, the Netherlands; Department of Family and Community Medicine, University of Toronto & Sport CARE, Women’s College Hospital, Toronto, Canada; Care to Move, St. Niklaas, Belgium; Aging, Rehabilitation & Geriatric Care Research Centre, Lawson Health Research Institute & Department of Family Medicine and School of Health Studies, Western University, London, Canada; Carbylan Therapeutics, Palo Alto, USA; Department of Epidemiology, University of Washington, Seattle, USA

**Keywords:** Knee Osteoarthritis, Hyaluronic acid, Triamcinolone acetonide, Viscosupplementation

## Abstract

**Background:**

Studies have evaluated the concomitant use of hyaluronan (HA) with steroids, anti-inflammatory drugs and analgesic agents in an attempt to magnify the extent and duration of pain relief due to knee osteoarthritis. To date there has not been an intra-articular combination therapy available for relief of knee osteoarthritis symptoms – one that combines the fast acting onset of symptom relief provided by a corticosteroid with the long-lasting symptom relief provided by HA in a single injection. The objective of this study was to evaluate the safety and preliminary performance of two new HA formulations, Hydros (hyaluronan-based hydrogel suspended in hyaluronan solution) and Hydros-TA (HA plus 10 mg of triamcinolone acetonide [TA]) in subjects with knee osteoarthritis.

**Methods:**

We conducted a Phase 2 prospective, multicenter, randomized, double-blind feasibility trial. Participants (n = 98; mean age 60 years) with knee osteoarthritis (Kellgren-Lawrence grade 2 or 3) were randomized to three treatment groups: Hydros, Hydros-TA, and Synvisc-One® (hylan G-F 20). Participants received one 6 ml intra-articular injection in the treatment knee and were evaluated at 2, 6, 13, and 26 weeks post-treatment. Safety was assessed from adverse events at all study visits. The primary efficacy outcome was the change from baseline on the Western Ontario and McMaster Universities Osteoarthritis Index (WOMAC) A (Pain) score for the treatment knee.

**Results:**

Adverse events were similar across treatment groups with the most common being arthralgia, joint swelling, back pain, and joint stiffness. Arthralgia was reported 5 times with Synvisc-One, 4 with Hydros, and 1 with Hydros-TA. Each group demonstrated a reduction in the WOMAC A (Pain) score over 26 weeks: [least-square mean (standard error)] 30.5 (5.1) mm for Hydros; 34.4 (4.7) mm for Hydros-TA; 28.0 (5.4) mm for Synvisc-One and an observed improvement of 2.5 mm (*p* = 0.64) and 6.4 mm (*p* = 0.24) over Synvisc-One for Hydros and Hydros-TA, respectively.

**Conclusions:**

A single injection of Hydros or Hydros-TA was well-tolerated and relieved pain associated with knee osteoarthritis over 26 weeks. Data indicate that Hydros-TA had a more rapid pain relief compared to Hydros alone. A Phase 3 trial is underway to confirm these preliminary results.

**Trial registration number:**

NCT01134406.

## Background

Osteoarthritis (OA) is a common degenerative joint disease that is characterized by loss of articular cartilage, subchondral bone sclerosis, osteophyte formation, changes in the synovial membrane, and reduced viscosity of synovial fluid [1]. OA is the leading cause of disability in high-income countries. On a global scale, OA is considered to be the fifth leading cause of Years Lost to Disability (YLD) in high-income countries, according to a World Health Organization (WHO) report [2]. Pain from OA has a major influence on a patient’s quality of life and carries a heavy economic burden.

There are several non-surgical options for treating pain associated with OA, ranging from measures that limit weight impact on the joints, to oral analgesics and anti-inflammatory drugs, to intra-articular (IA) steroid and viscosupplement injections [3].

HA is a linear polymer of repeating disaccharide units (each composed of D-glucuronic acid and D-N-acetylglucosamine, linked together via glycosidic bonds) and is found in significant concentration in the synovial fluid and articular cartilage of joints [4]. HA is responsible for the viscoelastic properties of synovial fluid and has been shown to be chondroprotective in experimental studies [5,6]. IA injections of HA are used for viscosupplementation of osteoarthritic joints to improve the viscoelastic properties of synovial fluid and relieve pain. In a meta-analysis of 54 randomized controlled trials, HA injections showed a therapeutic effect at 4 weeks post-injection which peaked at 8 weeks and extended over 6 months [7].

Viscosupplementation with HA is a common IA therapy that is effective in relieving knee pain caused by OA. However, viscosupplements typically have a delayed onset of pain relief, with little effect observed for the first 2 weeks following injection. A review by Bellamy et al. [8] involving 76 randomized, controlled trials confirmed that viscosupplements did not reach peak effectiveness until 5 weeks post-injection.

TA is a well-known synthetic corticosteroid, approved for IA injection, and is indicated as adjunctive therapy for short-term administration for acute episodes or exacerbations of OA. It is known from the literature that TA can directly inhibit the activation of inflammatory cells and their release of degradation products and recruitment factors [9]. A meta-analysis of 10 randomized controlled trials concluded that evidence supports short-term (up to two weeks) improvement in symptoms of knee OA after IA corticosteroid injection [10], and repeated use of IA corticosteroid injections has been found to be safe and clinically effective when given every three months over a period of two years [11].

TA is a well-known, well-studied synthetic corticosteroid that also has a long history of safe use for IA injection, having been on the US market for over 50 years. As different formulations, it is administered by injection, orally, by inhalation, and topically. It was first approved by the FDA in 1960. Corticosteroids are common IA therapies, and doses ranging from 10–40 mg have been demonstrated to be safe and effective for IA injection. In contrast to viscosupplements, corticosteroids have a very fast onset of pain relief but the effect is short-term, lasting 2 to 3 weeks. A Cochrane review which included 28 trials and 1,973 participants concluded that IA steroids were effective for 2 to 3 weeks post injection, but that there was a lack of evidence of effect on pain and function at 4 to 24 weeks post-injection [12]. Bannuru et al., had similar findings [13].

Studies have evaluated the concomitant use of HA with steroids, anti-inflammatory drugs, and analgesic agents (although they were not co-formulated) in an attempt to magnify the extent and duration of pain relief. In a one-year study of HA with and without TA, both groups improved, with the combination-therapy subjects improving sooner [14]. In addition, next to a fast-acting onset of symptom relief, corticosteroids may reduce the number of post-injection flares. Other clinical studies have successfully used steroids with HA for pain relief and consider this combination treatment as complementary therapy [15,16]. To date there has not been an IA combination therapy (co-formulated) available for relief of knee OA symptoms – one that combines the fast acting onset of symptom relief provided by a corticosteroid with the long-lasting symptom relief provided by HA in a single IA injection.

The purpose of this study was to evaluate the safety and preliminary performance of two new formulations, one HA product (Hydros) and one HA product that also contained a low-dose corticosteroid (Hydros-TA) in subjects with knee OA. These products were compared to Synvisc-One, a viscosupplement currently manufactured and distributed by Genzyme (a Sanofi company).

## Methods

### Materials

A new viscosupplement formulation, containing a biosresorbable HA-based hydrogel suspended in a HA solution, (Hydros), was developed to provide long-term relief from OA pain in a single IA injection. The hydrogel component is manufactured by crosslinking bacteria-derived, modified HA polymer with a polyethylene glycol crosslinker. A unique feature of Hydros is the ability to entrap a low-dose corticosteroid, in this case TA, into the hydrogel component. The addition of a corticosteroid (Hydros-TA) was designed to provide more rapid pain relief to the long lasting pain relief of Hydros alone. A single dose Hydros-TA contained 10 mg of TA.

These new formulations, Hydros and Hydros-TA, were compared to a control viscosupplement, Synvisc-One (hylan G-F 20), manufactured by Genzyme.

### Study population

Subjects were included in the study if they had knee OA of grades 2 or 3 on the Kellgren-Lawrence [17] grading system (confirmed radiologically), were ambulatory, and were between the ages 40 to 80 years old. Additionally, all subjects were required to have a WOMAC A (Pain) score averaging 50-90 mm in the treatment knee, using a visual analog scale (VAS) that ranged from 0-100 mm [18].

Subjects were excluded if they had: a WOMAC A (Pain) score >30 mm in the non-treated knee; IA steroid injections within the previous 3 months of the screening visit; IA HA injections within the 6 months prior to the screening visit; secondary OA or general symptomatic OA of lower extremity; hypersensitivity to avian proteins, corticosteroids, or HA-based products; active infection in either knee joint; uncontrolled diabetes; or a body mass index over 35.

All enrolled subjects had to agree to restrict pain medication to a maximum of 4 grams of paracetamol for knee pain (all other pain medication was excluded with the exception of NSAIDS for up to three days for non-treatment knee pain). No pain medication was allowed within 48 hours of a study visit.

### Study design

This Phase 2 trial was a prospective, multicenter, randomized, double-blind, feasibility study to evaluate the safety and performance of Hydros and Hydros-TA in subjects with knee OA (see Table 1 for participating centers). Subjects were equally randomized into one of three treatment groups and received a one-time 6 mL injection of Hydros, Hydros-TA, or Synvisc-One. Subjects were seen for post-injection follow-up assessment at 2, 6, 13, and 26 weeks.

**Table 1:** **Participating centers, Principal Investigators and number of subjects treated**

**Investigational site**	**Principal investigator**	**Total Pt.**
Lawson Health Research Institute Ontario, Canada	Robert Petrella, MD	39
Women's College Hospital Toronto, Canada	Julia Alleyne, MD	10
The University of British Columbia (UBC) Vancouver, Canada	Don McKenzie, MD	1
Sentro di Speshalista Spectrum Willemstad, Curacao	E. de Windt, MD	23
Research Orhopedie UZ Leuven, Belgium	Johan Bellemans, MD	2
Care to Move St. Niklaas, Belgium	Frank Dellaert, MD	11
University Hospital Maastricht Maastricht, The Netherlands	Pieter Emans, MD	11
St. Anna Ziekenhuis Hospital Geldrop, The Netherlands	H. J. Hoekstra, MD	1
**Eight (8) Enrolled Centers**	**Total:**	**98**

Subjects and evaluating physicians, who followed subjects post-treatment, were blinded to treatment. An injecting physician delivered the randomized treatment and remained unblinded. All randomized subjects received one IA injection in the treatment knee by an injecting physician. The treatment knee was the knee that met the inclusion criteria on the WOMAC A (Pain) score and received one of the three IA injection treatments, as determined by sequential randomization at each study site. The randomization treatment was computer generated and was stratified by study center.

The study was performed in strict adherence to the protocol and in accord with the Declaration of Helsinki, the applicable guidelines for good clinical practices, ISO 14155, Clinical Investigation of Medical Devices for Human Subjects, mandates of the Ethics Committees, and all applicable national and local laws and regulations. In addition, appropriate training and support in the use of the investigational product were provided. Investigators were responsible for enrolling into the study only those subjects who conformed to the inclusion criteria and for whom no exclusion criteria applied (study registered with ClinicalTrials.gov; NCT 01134406). Institutional review board and/or ethics committee review and approval was sought and obtained by each individual site. For example, at the largest site, approval was obtained from Western University’s Health Sciences Research Ethics Board. All subjects provided written informed consent.

### Safety evaluations

Assessments of all adverse events (AEs) for all study visits were performed by the blinded evaluating physician. Any AE reported post-treatment was considered a Treatment-Emergent Adverse Event (TEAE) and were summarized by treatment group, using a Medical Dictionary for Regulatory Activities (MedDRA) version 13.1 preferred terms, system organ classifications, and severity. If a subject experienced multiple events that mapped to a single preferred term, the greatest severity grade and strongest investigator assessment of relation to study medication was assigned to the preferred term for the appropriate summaries. For subjects who experienced the same coded event more than once, only one event was presented. AEs were assessed for relation to treatment and procedure.

### Primary efficacy assessment

The change from baseline in WOMAC A (Pain) score for knee pain covering the 48-hour period prior to the follow-up visit for all follow-up visits was measured for each subject.

### Secondary efficacy assessments

Changes in WOMAC B (Stiffness) and WOMAC C (Function) scores from baseline were evaluated similarly to the Pain score. Global assessments by the subjects at 13 weeks and subject and physician global assessments at 26 weeks were obtained. OMERACT-OARSI guidelines [19] were used to define the number and percentage of positive “strict responders” (≥50% and ≥20 mm improvements in WOMAC A (Pain) or C (Function) scores over baseline). Average weekly consumption of paracetamol in grams over the 26-week post-treatment period was captured.

### Statistical analyses

A sample size of 90 subjects (30 subjects per group) was intended to provide 1) preliminary information on the safety and tolerability of Hydros and Hydros-TA and 2) a preliminary review of efficacy of these two modalities as compared to the control, Synvisc-One. Intent-to-Treat (ITT) methodology (all randomized subjects) was used to analyze subjects by treatment group for efficacy and safety. For the primary efficacy endpoint, Least Square Means from a repeated measure ANCOVA model with effects for study center, treatment, visit, visit by treatment interaction, and baseline subscale value were utilized to assess difference between treatment groups. The Cochran-Mantel Haenszel General Association statistic, stratified by study center, was used to assess differences among treatments and to assess differences in the pairwise comparisons between treatments in the OMERACT-OARSI response rate. Rounding for all numerical values occurred only as the last step for all results presented.

## Results

### Participant flow and characteristics

**Figure 1:**
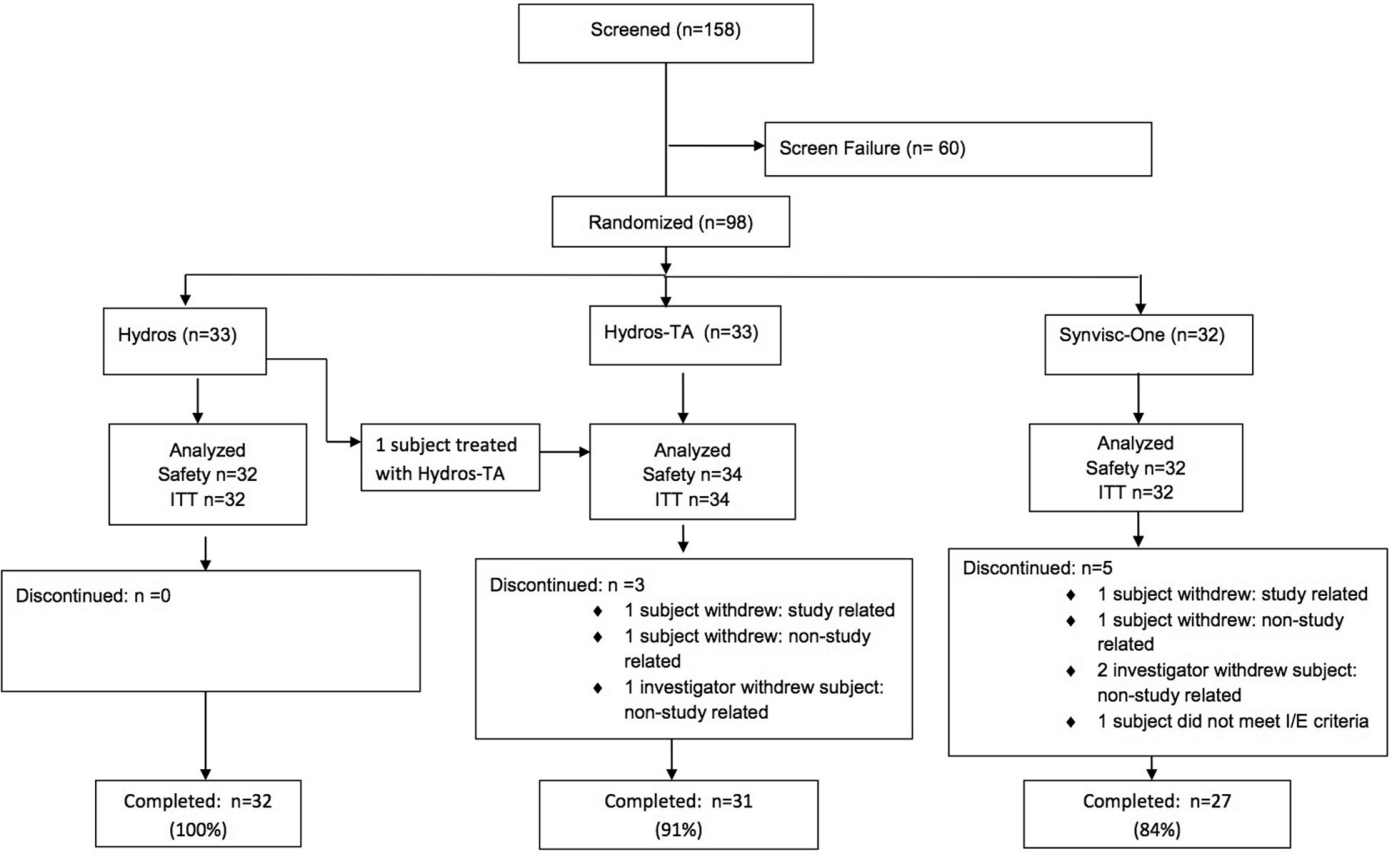
**COR 1.0 randomization and follow-up.**

Ninety-eight subjects out of 158 subjects screened met the eligibility criteria and were randomly assigned to one of three treatment groups (see Figure 1).

Demographics and OA knee baseline characteristics were similar between all groups (Table 2). The average subject age was 60 (range 40–84 years) and 43% were male. The Hydros group had the largest female to male ratio (20:12, 62%:38%). BMI was similar across all groups, and 76% of subjects were Caucasian.

**Table 2:** **Demographics and baseline characteristics by treatment group**

**Category**	**Hydros n = 32**	**Hydros TA n = 34**	**Synvisc-One n = 32**
**Age in years, mean (SD)**	59 (12)	61 (11)	59 (12)
**Gender, n (%)**			
**Male**	12 (38)	14 (41)	16 (50)
**Female**	20 (63)	20 (59)	16 (50)
**Race, n (%)**			
**Caucasian**	23 (72)	28 (82)	23 (72)
**Non-Caucasian**	9 (28)	6 (18)	9 (28)
**Body Mass Index in kg/m** ^**2**^ **, mean (SD)**	29.8 (4.1)	29.0 (4.1)	29.0 (3.8)
**Kellgren-Lawrence Grade for Treatment Knee, n (%)**			
**Grade 2**	18 (56)	22 (65)	18 (56)
**Grade 3**	14 (44)	12 (35)	14 (44)
**Pre-Treatment WOMAC score in mm, mean (SD)**			
**Treatment Knee**	68 (9)	69 (11)	66 (12)
**Non-treatment Knee**	13 (8)	12 (9)	12 (8)
**Duration of Symptoms - Pre-Treatment in Treatment Knee in months, mean (SD)**	64 (59)	74 (80)	69 (56)

Abbreviations: WOMAC = Western Ontario and McMaster Universities Osteoarthritis Index.

### Adverse events

A total of 7 subjects reported Serious Adverse Events (SAEs). In the Hydros group, the 3 reported SAEs were colitis, broncho-pneumonia and arthralgia. In the Hydros-TA group, the 2 reported SAEs included a report of a meniscal lesion and a cyst aspiration. Lastly, the two SAEs reported in the Synvisc-One group were a meniscal lesion and an elective surgery. All SAEs were considered unlikely or definitely not related to treatment and had resolved by the termination of the study.

Treatment Emergent Adverse Events (TEAEs) were reported in 69% of all subjects treated. The most commonly reported TEAEs were arthralgia, joint swelling, joint stiffness and back pain. Table 3 lists the AEs by treatment group, which occurred in 5% or more of the overall ITT population.

**Table 3:** **Number of adverse events reported in ≥ 5% of subjects by treatment group**

**AE description**	**Synvisc-One n = 32**	**Hydros n = 32**	**Hydros-TA n = 34**
**Any TEAE, n (%)**	23 (72)	24 (75)	21 (62)
**Arthralgia, n (%)**	8 (25)	11 (34)	7 (21)
**Joint Swelling, n (%)**	2 (6)	3 (9)	2 (6)
**Joint Stiffness, n (%)**	2 (6)	3 (9)	0
**Back Pain, n (%)**	3 (9)	1 (3)	1 (3)
**Muscle Spasms, n (%)**	1 (3)	0	3 (9)
**Arthritis, n (%)**	0	2 (6)	0
**Headache, n (%)**	8 (25)	6 (19)	4 (12)
**Nasopharyngitis, n (%)**	3 (9)	1 (3)	1 (3)
**Nausea, n (%)**	1 (3)	2 (6)	1 (3)
**Meniscus Lesion, n (%)**	1 (3)	0	2 (6)
**Tendon Pain, n (%)**	2 (6)	0	0

Abbreviations: AE = Adverse Event; TEAE = Treatment-Emergent Adverse Event.

AEs for 15 subjects were reported as “Likely” or “Definitely” related to study treatment: 6 in the Synvisc-One group, 6 in the Hydros group, and 3 in the Hydros-TA group. There were a total of 19 AEs related to study treatment in these 15 subjects: 9 in the Synvisc-One group, 7 in the Hydros group, and 3 in the Hydros-TA group. The most commonly reported AEs related to study treatment were arthralgia and joint stiffness. These results are summarized in Table 4.

**Table 4:** **Adverse events "likely" or "definitely" related to study treatment**

**System organ class**	**Preferred term**	**Synvisc-one n = 32**	**Hydros n = 32**	**Hydros-TA n = 34**
**Musculoskeletal and Connective Tissue disorders, n (%)**	**Arthralgia**	5 (16)	4 (13)	1 (3)
	**Joint Stiffness**	2 (6)	2 (6)	0
	**Joint Swelling**	1 (3)	0	0
**General Disorders and Administration Site conditions, n (%)**	**Application Site Warmth**	1 (3)	0	0
	**Injection Site Warmth**	0	0	1 (3)
	**Injection Site Movement Impairment**	0	1 (3)	0
	**Injection site pain**	0	0	1 (3)
**Total Related Adverse Events,** **n (%)**	**NA**	9 (28)	7 (22)	3 (9)

### Primary endpoint: WOMAC a (pain) score (ITT) population

For the WOMAC A (Pain) score change from baseline, the mean reductions from baseline over 26 weeks [Least-Square Means (LSMean) Standard Errors (SE)] were 30.5 (5.1) mm for Hydros, 34.4 (4.7) mm for Hydros-TA, and 28.0 (5.4) mm for Synvisc-One. These data as well as the mean reductions for WOMAC A (Pain) score for the ITT population for all three treatment groups at each post-treatment follow-up visit are shown in Table 5. Overall, Hydros and Hydros-TA mean reductions represent and observed improvement over Synvisc-One of 2.5 mm (p = 0.64) and 6.4 mm (p = 0.24), respectively.

**Table 5:** Mean reduction on WOMAC A (Pain), B (Stiffness), and C (Function) scores (mm)*

				**Between Group Differences**			
	**Mean reduction (Pain) from Baseline [LSMeans(SE)]**			**Synvisc-One – Hydros**		**Synvis-One – Hydros-TA**	
**Time Points**	**Synvisc-One n=32**	**Hydros n=32**	**Hydros-TA n=34**	**LSMeans**	***p*** **-value**	**LSMeans**	***p*** **-value**
Baseline	66.4	68.1	69.4	N/A	N/A	N/A	N/A
2 weeks	-28.5 (5.9)	-23.3 (5.6)	-35.6 (5.2)	5.2	0.40	-7.1	0.25
6 weeks	-25.6 (5.9)	-32.4 (5.6)	-33.4 (5.2)	-6.8	0.28	-7.8	0.21
13 weeks	-29.0 (6.0)	-33.9 (5.6)	-33.3 (5.2)	-4.9	0.43	-4.3	0.49
26 weeks	-28.9 (6.0)	-32.4 (5.6)	-35.2 (5.3)	-3.5	0.58	-6.3	0.33
Overall	-28.0 (5.4)	-30.5 (5.1)	-34.4 (4.7)	-2.5	0.64	-6.4	0.24
				**Between Group Differences**			
	**Mean reduction (Stiffness) from Baseline [LSMeans(SE)]**			**Synvisc-One – Hydros**		**Synvis-One – Hydros-TA**	
**Time Points**	**Synvisc-One n=32**	**Hydros n=32**	**Hydros-TA n=34**	**LSMeans**	***p*** **-value**	**LSMeans**	***p*** **-value**
Baseline	70.1	70.5	70.3	N/A	N/A	N/A	N/A
2 weeks	-25.9 (6.7)	-18.4 (6.4)	-33.7 (6.0)	7.5	0.29	-7.8	0.27
6 weeks	-24.0 (6.7)	-24.2 (6.4)	-31.4 (6.0)	-0.2	0.98	-7.4	0.30
13 weeks	-25.9 (6.8)	-26.4 (6.4)	-27.4 (6.0)	-0.5	0.94	-1.5	0.83
26 weeks	-24.6 (6.9)	-29.0 (6.5)	-26.8 (6.0)	-4.4	0.55	-2.2	0.77
Overall	-25.1 (6.1)	-24.5 (5.8)	-29.8 (5.3)	0.6	0.92	-4.7	0.43
				**Between Group Differences**			
	**Mean reduction (Function) from Baseline [LSMeans(SE)]**			**Synvisc-One – Hydros**		**Synvis-One – Hydros-TA**	
**Time Points**	**Synvisc-One n=32**	**Hydros n=32**	**Hydros-TA n=34**	**LSMeans**	***p*** **-value**	**LSMeans**	***p*** **-value**
Baseline	63.5	66.2	65.1	N/A	N/A	N/A	N/A
2 weeks	-23.7 (6.0)	-20.2 (5.8)	-32.1 (5.3)	3.5	0.58	-8.4	0.18
6 weeks	-23.5 (6.0)	-29.2 (5.8)	-29.0 (5.3)	-5.7	0.37	-5.5	0.38
13 weeks	-25.6 (6.1)	-31.0 (5.8)	-29.1 (5.3)	-5.4	0.39	-3.5	0.58
26 weeks	-24.5 (6.1)	-29.0 (5.8)	-29.5 (5.4)	-4.5	0.49	-5.0	0.44
Overall	-24.3 (5.6)	-27.3 (5.4)	-30.0 (4.9)	-3.0	0.59	-5.6	0.31

*For the intent-to-treat population in the Synvisc-One, Hydros, and Hydros TA groups, observed at landmark time points.

Abbreviations: WOMAC = Western Ontario and McMaster Universities Osteoarthritis Index; LSMeans = Least Squares Means; SE = Standard Error.

### Secondary endpoint: WOMAC B (stiffness) score

The mean changes in WOMAC B (Stiffness) score from baseline for the ITT population for each treatment group are 24.5 (5.8) mm for Hydros, 29.8 (5.3) mm for Hydros-TA, and 25.1 (6.1) mm for Synvisc-One. The complete mean reduction data for the WOMAC B (Stiffness) score are reported in Table 5.

### Secondary endpoint: WOMAC C (function) score

Hydros, Hydros-TA, and Synvisc-One had reductions of 27.3 (5.4) mm, 30.0 (4.9) mm, and 24.3 (5.6) mm, respectively, for the changes from baseline of the overall mean WOMAC C (Function) score. The complete results for mean reduction of WOMAC C (Function) score in the ITT population are shown in Table 5.

In addition, a comparison of the pain reduction provided by Hydros-TA versus Hydros is presented in Table 6. While there was not a statistically significant difference between Hydros-TA and Hydros over 26 weeks, there was a 12.4 mm improvement in pain scores at the 2 week time point for Hydros-TA vs. Hydros (p = 0.04). These results confirm the earlier onset of pain relief for the steroid containing therapy (Hydros-TA) versus the non-steroid containing therapy (Hydros).

**Table 6:** Hydros-TA vs. Hydros: Observed mean reduction in WOMAC A (Pain) score*

**Time point**	**Hydros n = 32**	**Hydros-TA n = 34**	**Difference**	***p*** **-value**
	**Mean reduction (mm) from baseline [LSMeans(SE)]**		**Estimated between group difference [LSMeans(SE)]**	
Baseline	68.1	69.4	NA	NA
2 weeks	−23.3 (5.6)	−35.6 (5.2)	−12.4	0.04
6 weeks	−32.4 (5.6)	−33.4 (5.2)	−1.1	0.86
13 weeks	−33.9 (5.6)	−33.3 (5.2)	0.6	0.93
26 weeks	−32.4 (5.6)	−35.2 (5.3)	−2.8	0.65
Overall	−30.5 (5.1)	−34.4 (4.7)	−3.9	0.45

*For intent-to-treat population observed at landmark time points.

### Secondary endpoint: strict OMERACT-OARSI responder analysis

The percentages of strict OMERACT-OARSI responders for Hydros and Hydros-TA were numerically higher than the strict responders for Synvisc-One at 13 and 26 weeks (see Table 7).

**Table 7:** **Strict OMERACT-OARSI responder analysis**

**Group**	**13 Weeks n (%)**	**26 Weeks n (%)**
**Synvisc-One**	17 (53)	19 (59)
**Hydros**	21 (66)	22 (69)
**Hydros-TA**	24 (71)	22 (65)

## Discussion

This study demonstrated the preliminary safety and tolerability of two new formulations, Hydros and Hydros-TA, for the treatment of knee OA. Additionally, the results of the WOMAC A (Pain) and WOMAC C (Function) scores, for both the Hydros and Hydros-TA, and the WOMAC B (Stiffness) score for Hydros-TA showed the potential for improved outcomes when compared to Synvisc-One over the 26 weeks studied. As anticipated in this pilot feasibility study and due to the small sample size, the improvements following treatment with Hydros and Hydros-TA were not statistically significantly different from improvements with Synvisc-One, but the observed pain reduction with Hydros-TA was consistently greater than with Synvisc-One. Furthermore, the trend seen in the observed pain reduction in Hydros-TA compared to an active comparator (Synvisc-One) was approximately 10% from baseline and provides compelling evidence to move forward with a more confirmatory Phase 3 trial.

To our knowledge, this is the first Phase 2 randomized trial to report on the safety and preliminary efficacy of a single-injection viscosupplement that combines HA and corticosteroid for the treatment of knee OA. Reductions in WOMAC A (Pain) scores observed in this study suggest that this combination may have a faster onset of pain relief compared to non-steroid containing products and may provide improved pain relief over the full 26 weeks following injection. These results demonstrate the possible synergistic effect of combining HA with a corticosteroid. Ozturk et al. [14] in a similar patient population demonstrated faster and better pain relief when 2 injections of TA were added to a 6-injection HA protocol. Grecomoro et al. [16] also showed better pain relief in knee OA patients at earlier time points when a corticosteroid was added to the first of 5 HA injections. Both of these studies support the observations of the present study.

Hydros-TA is a proprietary formulation that enables the delivery of a corticosteroid that is physically trapped within the viscosupplement. The results obtained with Hydros-TA described above suggest that combining corticosteroids and HA in a single injection provides more rapid and sustained pain relief.

All three treatments were well tolerated. The total number of AEs among treatment groups was very similar; however, the numbers of AEs considered by the treatment-blinded evaluating physicians to be ‘likely’ or ‘definitely’ related to the study treatment were higher in the Synvisc-One and the Hydros groups, as compared to the Hydros-TA group. In addition, the percentage of subjects who responded favorably to the product (as measured by the OMERACT-OARSI responder rate) was higher in Hydros and Hydros-TA groups as compared to Synvisc-One.

Inferences related to this study are limited by the small number of subjects in each group. However, the trends of improved pain relief observed at all time points in the Hydros-TA arm compared to Synvisc-One arm indicate that future Phase 3 trials are warranted to evaluate the effects of Hydros with the addition of corticosteroids for patients with knee OA.

## Conclusions

The present study demonstrated that a single injection of Hydros or Hydros-TA was well-tolerated and relieved pain associated with knee OA over 26 weeks. The study endpoints indicate that Hydros-TA provides enhanced pain relief compared to Synvisc-One. Of particular note, the data from this study suggest that Hydros-TA had a more rapid pain relief compared to the hyaluronic acid formulations alone. A Phase 3 trial is currently underway to confirm these preliminary results.
